# Association of gene polymorphisms with women urinary incontinence

**DOI:** 10.1515/med-2021-0332

**Published:** 2021-08-25

**Authors:** Povilas Aniulis, Aurelija Podlipskyte, Alina Smalinskiene, Rosita Aniuliene, Mindaugas Jievaltas

**Affiliations:** Urology Clinic, Medical Academy, Lithuanian University of Health Sciences, A. Mickeviciaus Str. 9, Kaunas LT-44307, Lithuania; Laboratory of Behavioral Medicine, Neuroscience Institute, Lithuanian University of Health Sciences, Palanga LT-00135, Lithuania; Institute of Biology Systems And Genetic Research, Lithuanian University Of Health Sciences, Kaunas LT-44307, Lithuania; Obstetrics And Gynecology Clinic, Medical Academy, Lithuanian University of Health Sciences, Kaunas LT-44307, Lithuania

**Keywords:** ADRB3, HTR2A, serotonin receptor 2A, gene polymorphism, UI

## Abstract

Aim of study was set to investigate the association of women urinary incontinence (UI) with serotonin receptor *HTR2A* T102C and beta 3-adrenergic receptor *ADRB3* Trp64Arg genes polymorphisms. The study included 110 women with Urge, Stress, and Mixed UI types and the control group – 105 continent women. Both groups have filled in the ICIQ-FLUTS questionnaire and their blood genotyping was performed. Urge UI subgroup was older and had higher body mass index (BMI) in comparison to other UI types and control group. More than half of all women had family history of UI in Stress UI and Mixed UI subgroups. The frequency of *HTR2A* T102C gene polymorphism’s minor allele C and genotype CC was significantly more expressed in Urge UI subgroup, as compared with control group (C-77.3 vs 58.7%, *p* = 0.007 and CC-57.6 vs 31.1%, *p* = 0.015). The *ADRB3* Trp64Arg gene polymorphism did not differ between groups. The regression analysis revealed CC genotype (OR = 3.06, 95% CI: 1.11–8.43; *p* = 0.030) and allele C (OR = 2.53, 95% CI: 1.16–5.53; *p* = 0.020) were risk factors for development of Urge UI. We conclude that *HTR2A* T102C gene polymorphism affected the development of Urge UI.

## 1 Introduction

Urinary incontinence (UI) is the involuntary flow of urine due to a disruption of the normal urination mechanism, causing medical, social, and hygienic problems. For a woman suffering from this disease, the quality of life deteriorates very sharply [1,2]. The most common types of UI in women, according to consensus definitions developed by the International Urogynecological Association/International Continence Society, include stress urinary incontinence (SUI), mixed urinary incontinence (MUI), and urge urinary incontinence (UUI) [3]. UUI is a major symptom in the diagnosis of overactive bladder syndrome (OAB) [4].

Age is probably the best-known risk factor in the etiology of UI and the incidence of UI correlates directly with age. Approximately every 5–6th of 30-year-old female patients, about every second 50-year-old female patients, and around 80% of elderly women of 80 years old suffer from at least one type of UI [5,6,7]. A number of risk factors, such as lower estrogen levels in the vagina, bladder, and urethra during menopause, BMI > 30 kg/m^2^ [8], number of pregnancies [9,10], obstetrical events, at least one vaginal birth (much higher risk than delivery by cesarean) [11,12], frequent use of caffeine, smoking [13], and constipation [14], and neurological diseases such as stroke, multiple sclerosis, or Parkinson’s disease [15] are known as significant for the development of different types of UI.

It is known that family history of UI could be a risk factor for development of UI in women. The results from population-based Norwegian EPICONT study showed 1.3-fold greater risk for UI if mother had UI and 1.6-fold risk if an older sister had UI [16].

Ethnicity is also considered as one of the risk factors for UI. African-Americans are more likely to suffer from UUI (45.9%) than white (43.4%), hispanic (42%), and Asian-born women (26.6%) [13]. Heritability of 34–41% for UI was determined in monozygotic and dizygotic twin studies [17,18].

These findings support the hypothesis about genetic basis toward developing of UI.

There are some clinical studies that reported associations among female with UI and genetic factors, particularly in single-nucleotide polymorphism (SNP) [19,20].

Researchers Penney et al. evaluated 6120 women and found that 68 SNP, located in 2 loci, chromosomes 8q23.3 and 1p32.2, were significantly associated with UI [21].

However, the evidence on SNPs in UI is still scarce. It is known that urinary function is controlled by the central and peripheral nervous systems through various neurotransmitters [15].

The SNP of the beta 3-adrenoceptor *(ADRB3)* Trp64Arg gene polymorphism or rs4994 is a mutation of the thymine at position 190 to cytosine and changes protein receptor in 64th position of tryptophan to arginine [22]. The SNP (rs4994) of *ADRB3* gene has been extensively studied in obesity, type-2 diabetes, and other diseases pathogenesis and found that *ADRB3* expression in bladder cells has been shown to be very high and promotes muscle relaxation [20].

In the human bladder, the detrusor muscle is mainly released through the activation of *ADRB3* [23,24]. Recent studies suggest that activation of *ADRB3* in urothelium and bladder detrusor muscle may have an inhibitory effect on afferent bladder activity, suggesting that *ADRB3* affects bladder sensitivity [25,26].

It is also thought that SNP of the *ADRB3* gene may lead to incomplete detrusor relaxation during the bladder filling phase. As a result, it is thought that this polymorphism may contribute to the development of UUI.

Serotonin (5-hydroxytryptamine; 5-HT) is a monoamine transmitter that is primarily found in the gastrointestinal tract, platelets, and brain. 5-HT causes smooth muscle contraction in various tissues such as the intestine, arteries, and bladder [27].

The serotonin 2A receptor gene *(HTR2A*) is located on 13 chromosome (13q14-21) and consists of three exons spanning >20 kb.

For these reasons, we hypothesized that SNP in these two genes may have a significant association with bladder function and the development of UI. Identification of genetic factors, associated with UI, could result to a better understanding of etiologic pathways and would allow planning the new treatment strategies.

The aim of our study is to investigate the association of women UI with serotonin receptor (*HTR2A*) T102C and beta 3-adrenergic receptor (*ADRB3*) Trp64Arg genes polymorphisms as a risk factor for the development of different types of UI.

## 2 Materials and methods

### 2.1 Ethics

The study protocol was approved by Biomedical Research Ethics Committee of Lithuanian University of Health Sciences (LUHS) (permission No. BE-2-50).

### 2.2 Study population and setting

This case control study included women from 40 to 70 years old, consecutively admitted for consultation to gynecology or urology outpatient departments at the Hospital of LUHS. Duration of the study was 24 months. All participants signed informed consent to participate in the study.

The exclusion criteria were more than two births, oncological history, diabetes mellitus, previous pelvic surgery, previous treatment of UI, persistent urinary tract infection, neurological diseases that can cause UI, history of mental disorders, and BMI higher than 35 m^2^/kg.

Sociodemographic and medical history were collected and International Consultation on Incontinence Questionnaire Female Lower Urinary Tract Symptoms (ICIQ-FLUTS) was filled in for evaluating female lower urinary tract symptoms [28]. Study subjects were classified to control group (continent women) and study group – subjects with different types of UI (SUI, UUI, and MUI). Women reporting no clinically significant symptoms of UI of any type (overall score ≤2 on ICIQ-FLUTS) were included into control group [29]. The final diagnosis of type of UI medical examination of gynecologist and urologist was performed – the ultrasound to evaluate residual urine, cough, and valsalva tests. In the cases of MUI, urodynamic evaluation (uroflowmetry and cystometry) was performed. Thus, all incontinent women were divided into three subgroups according to diagnostic criteria for different types of UI: SUI, wet and dry types of UUI, and MUI.

From 260 women, invited to participate in the study, 10 declined to participate, 22 had exclusion criteria, and three returned incomplete questionnaires. Ten genotyping was not performed due to sample contamination.

The final study sample consisted of 110 women (mean age 57.0 (9.3)), diagnosed with three types of UI, and 105 women (mean age 52.4 (8.2)) as healthy control group.

Total response rate was 82.7%. The venous blood was taken from all study participants and genotyping of *ADRB3* and *HTR2A* gene polymorphism was performed.

## 3 Assessments

### 3.1 The ICIQ-FLUTS questionnaire

ICIQ-FLUTS questionnaire consists of three subscales (12 questions) and it is completed in average of 4–5 min. All answers are based on experiences with LUTS in the previous four weeks. Each element of the question has two sections. The first section asks about the symptom and quantifies it on a Likert scale (0 – never to 4 – always). The second section identifies the extent to which this symptom bothers and disturbs subject (the score is calculated by domains and it ranges from 0 – there are no problems, to 10 – it is very troublesome).

The first subscale of the ICIQ-FLUTS is for the symptoms of bladder filling (four questions, sum of the scores from 0 to 16), the second – for the symptoms of voiding (three questions, sum is from 0 to 12), and the third – for the symptoms of incontinence (five questions, score is from 0 to 20). Higher scores indicate stronger symptoms. The different bladder filling, voiding, and incontinence scores represent different types of UI. In this study, we used the ICIQ-FLUTS, validated for use in Lithuanian language according to the author’s recommendations [30].

### 3.2 *ADRB3* and *HTR2A* genotyping

Genotyping of the *ADRB3* rs4994 and *HTR2A* rs6313 was done at the Laboratory of Genetics of the Institute of Biology Systems and Genetic Research of LUHS. Blood samples of DNA extraction were collected in ethylenediaminetetraacetic acid (EDTA) tubes. Genomic DNA from peripheral blood leucocytes was extracted using genomic DNA purification kit (Thermo Fisher Scientific, Waltham, MA, USA) according to the manufacturer’s recommendations. Single-nucleotide polymorphisms (SNPs in *ADRB3* gene (rs4994)) were estimated by using a commercially available genotyping kit C_2215549_20 and in *HTR2A* gene (rs6313) C_3042197_1_ (Applied Biosystems, Foster City, CA, USA). The Applied Biosystems 7900HT Real-Time Polymerase Chain Reaction System (Applied Biosystems, Foster City, CA, USA) was used for SNPs detection. The cycling program started with heating at 95°C for 10 min, followed by 40 cycles (at 95°C for 15 s and at 60°C for 1 min). Finally, allelic discrimination was done by using SDS 2.3 software provided by Applied Biosystems.

### 3.3 Statistical analysis

The clinical characteristics were reported by frequencies and percentages for the categorical variables and with means and standard deviations for the continuous variables. A *χ* ^2^ test is used to calculate the Hardy-Weinberg equilibrium (HWE), complied with the law of equilibrium (*p* > 0.05). Numeric data means were compared with the use of the parametric ANOVA test or nonparametric Kruskal–Wallis *K* tests. The four inheritance models (codominant, dominant, recessive, and over dominant) were applied in a logistic regression analysis to estimate the risk of UI between cases in UI subgroups and control group. Odds ratio (OR) with a 95% confidence interval (CI) was reported for allelic comparisons and genotype frequencies between the different UI type of subgroups and the control group. Logistic regression analysis was adjusted by age, family history for UI, and BMI. Statistical analyses were performed with the Statistical Package for the Science Software v.22 (SPSS, Chicago, IL). The level of significance was set at *p* < 0.05.

## 4 Results

The comparison of sociodemographic and clinical characteristics collected from medical history and ICIQ-FLUTS questionnaire was presented in Table 1.

**Table 1:** The comparison of sociodemographic and clinical data among women with UI and control group

Characteristics	Control group, *n* = 105	Women with UI (subgroups), *n* = 110			*p* value
		SUI, *n* = 55	UUI, *n* = 34	MUI, *n* = 21	
Age (years), mean ± SD	52.4 ± 8.2	53.0 ± 9.2	61.8 ± 7.3	59.6 ± 8.3	**<0.001**
					^1^0.676
					^2^<**0.001**
					^3^ **0.001**
BMI (kg/m^2^), mean ± SD	24.6 ± 4.0	26.0 ± 3.3	27.4 ± 4.0	25.6 ± 3.1	**0.001**
					^**1**^ **0.026**
					^2^ **0.001**
					^3^0.268
Family history for UI, *n* (%)	7 (6.7)	28 (50.9)	10 (29.4)	11 (52.4)	**<0.001**
					^1^ **<0.001**
					^2^ **0.001**
					^3^ **<0.001**
ICIQ-FLUTS, median (IQR)	1.0 (0.0–2.0)	11.0 (8.0–14.0)	19.0 (14.8–21.3)	23.0 (17.0–26.0)	**<0.001** ^**#**^
					^1^ **<0.001**
					^2^<**0.001**
					^3^ **<0.001**
Filling (score)	1.0 (0.0–2.0)	1.0 (0.0–3.0)	10.0 (8.0–11.0)	9.0 (5.5–10.0)	**<0.001** ^**#**^
					^1^ **0.007**
					^2^<**0.001**
					^3^ **<0.001**
Voiding (score)	0.0 (0.0–0.0)	1.0 (0.0–2.0)	0.0 (0.0–1.0)	1.0 (0.0–2.0)	**<0.001** ^**#**^
					^1^ **<0.001**
					^2^<**0.001**
					^3^ **<0.001**
Incontinence (score)	0.0 (0.0–0.0)	8.0 (7.0–10.0)	8.0 (5.0–11.0)	13.0 (11.0–15.0)	**<0.001** ^**#**^
					^1^ **<0.001**
					^2^<**0.001**
					^3^ **<0.001**

Difference between control group and different UI subgroups was evaluated by *χ* ^2^ and one-way ANOVA or nonparametric Kruskal–Wallis test^#^.

*p*^1^ Control group vs SUI, *p* ^2^ Control vs UUI, *p* ^3^ Control vs MUI.

Abbreviations: UI, urinary incontinence; SUI, stress urinary incontinence; UUI, overactive bladder; MUI, mixed urinary incontinence; BMI, body mass index; ICIQ-FLUTS, international consultation on incontinence modular questionnaire female lower urinary tract symptoms; SD, standard deviations; IQR, interquartile range (P25–P75).

In bold, *p* < 0.05.

Age and BMI were significantly higher in UUI subgroup, as compared to SUI, MUI subgroups, and study control group. There were more than half of women with family history of UI in SUI and MUI subgroups. In UUI subgroup, family history of UI was significantly lower than that in SUI and in MUI subgroups (29.4 vs 50.9%, respectively; *p* = 0.049 and 52.4%, respectively, *p* = 0.094), but significantly higher than that in control group (6.7%; *p* < 0.001).

The total score of the ICIQ-FLUTS questionnaire, reported higher severity of LUTS symptoms, was statistically significantly higher in UUI and MUI subgroups in comparison to SUI subgroup and healthy control group. The filling scores in UUI and MUI subgroups were significantly higher than SUI (*p* < 0.001); however, SUI did not differ from control group. The voiding score was significantly higher in all women with UI (*p* < 0.001), without significant differences among study subgroups. The incontinence score was significantly higher in MUI in comparison to other subgroups (*p* < 0.05) and control group; the severity of incontinence did not differ among SUI and UUI subgroups.

Allele frequencies and genotype distribution between the control group and SUI, UUI, and MUI subgroups are presented in Table 2. The frequency of *HTR2A* (rs6313) T102C gene polymorphism’s minor allele (C) was significantly more expressed in the UUI subgroup, as compared with control group (77.3 vs 58.7%, respectively, *p* = 0.007). The polymorphism of *HTR2A* genotype CC was statistically significantly more expressed in UUI subgroup patients, as compared with control group (57.6 vs 31.1%, respectively, *p* = 0.015); without differences in other UI subgroups. The allele frequencies and genotype distribution *of ADRB3* (rs4994) Trp64Arg gene polymorphisms did not differ statistically between the UI subgroups and study control group.

**Table 2:** Allele frequencies and genotype distribution among the control group (*n* = 105) and women with different types of UI (subgroups): SUI (*n* = 55), UUI (*n* = 34) and MUI (*n* = 21)

Polymorphism	Genotype/allele	Control group, *n* (%)	SUI, *n* (%)	UUI, *n* (%)	MUI, *n* (%)	*p* value
HTR2A (rs6313) C > T	Genotype					**0.037**
	CC	32 (31.1)	26 (49.1)	19 (57.6)	7 (33.3)	^1^0.073
	CT	57 (55.3)	20 (37.7)	13 (39.4)	9 (42.9)	^2^ **0.015**
	TT	14 (13.6)	7 (13.2)	1 (3.0)	5 (23.8)	^3^0.422
	Allele					**0.021**
	C	121 (58.7)	72 (67.9)	51 (77.3)	23 (54.8)	^1^0.115
	T	85 (41.3)	34 (32.1)	15 (22.7)	19 (45.2)	^2^ **0.007**
						^3^0.635
ADRB3 (rs4994) T > C	Genotype					0.693
	CC	1 (1.0)	0 (0.0)	1 (2.9)	1 (4.8)	^1^0.755
	TC	14 (13.3)	8 (14.5)	3 (8.8)	3 (14.3)	^2^0.562
	TT	90 (85.7)	47 (85.5)	30 (88.8)	17 (81.0)	^3^0.436
	Allele					0.810
	C	16 (8.1)	8 (7.3)	5 (7.4)	5 (11.9)	^1^0.911
	T	194 (91.9)	102 (92.7)	63 (92.6)	37 (88.1)	^2^0.943
						^3^0.360

*p*^1^ Control group vs SUI, *p* ^2^ Control group vs UUI, *p* ^3^ Control group vs MUI.

Abbreviations: UI, urinary incontinence; SUI, stress urinary incontinence; UUI, overactive bladder; MUI, mixed urinary incontinence.

In bold, *p* < 0.05.

The logistic regression analysis was performed to evaluate polymorphism as the risk factor for three types of UI (Table 3), considering the fact that SUI, UUI, and MUI subgroups and control group were not homogeneous (according to age, BMI, and family history of UI).

**Table 3:** *ADRB3* Trp 64 Arg and *HTR2A* T102C polymorphisms influence in different UI types

Polymorphism	Model	Genotype	Control vs SUI, OR (95% CI)	*p*-value	Control vs UUI, OR (95% CI)	*p*-value	Control vs MUI, OR (95% CI)	*p*-value
HTR2A (*rs6313*) C > T	Allele	T	1	0.226	1	**0.020**	1	0.351
		C	1.43 (0.80–2.56)		2.53 (1.16–5.53)		0.66 (0.28–1.57)	
	Dominant	CT + TT	1	0.063	1	**0.030**	1	0.688
		CC	2.2 (0.96–4.94)		3.06 (1.11–8.43)		0.77 (0.21–2.81)	
	Recessive	TT	1	0.640	—	—	1	0.228
		CT + CC	1.03 (0.92–1.14)				2.51 (0.56–11.24)	
	Codominant	CC	1		1		1	
		CT	0.38 (0.15–0.93)	**0.034**	0.39 (0.14–1.12)	0.081	0.95 (0.21–4.30)	0.949
		TT	0.81 (0.43–1.52)	0.519	—	—	1.31 (0.58–2.97)	0.523
	Over dominant		1	0.055	1	0.239	1	0.599
			0.45 (0.20–1.02)		0.56 (0.21–1.48)		0.73 (0.22–2.40)	
ADRB3 (*rs4994*) T > C	Allele	C	1	0.524	1	0.936	1	0.284
		T	1.41 (0.49–4.04)		0.95 (0.25–3.65)		0.49 (0.13–1.80)	
	Dominant	TT	1	0.793	1	0.974	1	0.378
		TC + CC	0.86 (0.29–2.58)		0.98 (0.21–4.54)		1.98 (0.44–8.98)	
	Recessive	TT + CC	—		—		—	
		CC						
	Codominant	TT	1	0.941	1	0.875	1	0.545
		TC	1.04 (0.35–3.13)		0.88 (0.17–4.59)		1.67 (0.32–8.87)	
		CC	—		—		—	
	Over dominant	TT + CC	1	0.875	1	0.870	1	0.559
		TC	1.09 (0.37–3.22)		0.87 (0.17–4.58)		1.63 (0.32–8.27)	

Age, family history for UI and BMI were introduced in the model as control variables.

Abbreviations: UI, urinary incontinence; SUI, stress urinary incontinence; UUI, overactive bladder; MUI, mixed urinary incontinence, OR, odd ratio.

In bold, *p* < 0.05.

The dominant model revealed CC genotype 3-fold increased risk for the development of the UUI (OR = 3.06, 95% CI: 1.11–8.43; *p* = 0.030). 2.5-fold increased risk of the development of UUI was found for every copy of allele C (OR = 2.53, 95% CI: 1.16–5.53; *p* = 0.020).

The codominant model revealed that CT genotype was an independent variable, decreasing risk for the development of SUI (OR = 0.38, 95% CI: 0.15–0.93; *p* = 0.034).

## 5 Discussion

Polymorphism of the *HTR2A* gene T102C (rs6313) is associated with the CNS serotonin metabolism system, which modulates bladder activity function [18]. In rats with partial bladder outflow obstruction, *HTR2A* receptors mRNA levels were increased in detrusor and subserosal layer of the bladder, but not in the urothelium. The increase in *HTR2A* receptor mRNA persisted from 3 to 14 days after induced obstruction. These results indicated that the induced partial urinary outflow obstruction caused an increase in the *HTR2A* receptor in the detrusor and subserosal layer of the bladder, which was later likely to result in forced bladder contraction [31].

We have found statistically significant difference in family history of UI between three evaluated types of UI and control group. This supports our hypothesis that UI may be related to genetic factors. The study of Ferreira et al. in 2011 has found that a family history of OAB is an independent risk factor for UUI and OAB syndrome [32]. However, our study showed opposite results: family history for UI was statistically significantly lower in the UUI subgroup compared with SUI and MUI subgroups. To explain the reason of that, our study result is controversial.

The next finding in our study is the association between *HTR2A* gene T102C polymorphism and development of UUI. It is known that the relation between *HTR2A* gene polymorphism and UI was evaluated in Brazilian study and has found the significant correlation between TT genotype and UI: subjects with the TT genotype had a 2-fold greater risk for UI than subjects with other genotypes [20]. Noronha et al.’s study has reported similar association between the TT genotype versus CC + CT genotypes and UI (OR = 2.69; 95% CI: 1.37–5.29; *p* = 0.013). And study group participants with the TT genotype were more prone to UUI, but participants with CC and TC genotype for SUI [33].

Our study demonstrated that the association of *HTR2A* gene (rs6313) T102C polymorphism’s minor allele (C) was statistically significantly more common in the UUI subgroup compared to control group and dominant model showed that CC genotype has 3-fold higher risk for the development of an UUI; 2.5-fold increase of risk of the development of UUI was found for every copy of allele C. These results appear to be in contrast to the aforementioned studies, perhaps this was due to the fact that women with fewer risk factors were included in our study.

Our study did not show significant associations between *ADBR3* gene Trp64Arg polymorphism and any type of UI; however, other studies showed different results.

*ADBR3* gene polymorphisms and UI were evaluated in the study of Honda et al. They included 100 women with UUI and OAB and 101 of healthy control group; DNA from hair root samples were obtained for *ADRB3* gene analysis. This study showed that overall frequency of the 64Arg variant (heterozygous plus homozygous) in patients with UUI was 47% and significantly higher than the frequency of 22.8% found in control group. The authors concluded that the Trp64Arg polymorphism in the *ADBR3* gene is weakly but significantly associated with UUI and OAB syndrome [34]. In the Brazilian study, scientists have investigated genetic polymorphisms in women with UUI and OAB syndrome. There were digested homozygous T allele 69.75%, heterozygotes 29.8%, and homozygous A allele 0.45%. A comparison between the groups showed higher prevalence of the digested homozygous T allele genotype in women with UUI and OAB syndrome. Family history of OAB syndrome was evaluated as an independent risk factor for UUI and OAB syndrome. This study concluded that the Trp64Arg polymorphism was associated with UUI and OAB syndrome in the Brazilian population [32]. These two studies were included in meta-analysis done by Cartwright et al. (2015), which concluded that the Trp64Arg (rs4994) polymorphism of the *ADRB3* gene was associated with UUI and OAB (OR = 2.5; 95% CI: 1.67–3.60; *n* = 419), with no heterogenity [19].

Despite the fact that we did not obtain statistically significant findings in the *ADRB3* gene polymorphism in different types of UI, an assessment of previous research findings, as well as strong association with a family history in UUI and OAB, we could suggest that UI has a genetic impact. The limitation of our study is the relatively small sample size to make the strong conclusion about significant genetic associations.

The strength of our study is the strict selection of the study sample, medical examination, and use of ICIQ-FLUTS questionnaire for clinically approved diagnosis of three types of UI. To our knowledge, this is the first study evaluated that concluded that the *HTR2A* gene T102C polymorphism showed a significant association with the development of UUI among women in Lithuanian population. Women with the CC genotype of the *HTR2A* gene are more prone to UUI, so we believe that in order to minimize the onset of this disease in life, these women should avoid and reduce other risk factors for this disease.
